# Nicotine from a Different Angle: Biological Effects from a Psychoneuroimmunological Perspective

**DOI:** 10.3390/ijms26136437

**Published:** 2025-07-04

**Authors:** Liudas Vincentas Sinkevicius, Sandra Sakalauskaite, Maris Bukovskis, Margus Lõokene, Vahur Valvere, Brigita Gradauskiene, Margus Viigimaa

**Affiliations:** 1 Department of Health Psychology, Lithuanian University of Health Sciences, 50161 Kaunas, Lithuania; liudas.vincentas.sinkevicius@lsmu.lt; 2 Institute of Psychology, Mykolas Romeris University, 08303 Vilnius, Lithuania; 3 Laboratory of Immunology, Department of Immunology and Allergology, Lithuanian University of Health Sciences, 50161 Kaunas, Lithuania; 4 Faculty of Medicine, University of Latvia, 1586 Ryga, Latvia; 5 Toompargi Vaimse Tervise Keskus, 10149 Tallinn, Estonia; 6 Clinic of Oncology and Hematology, North Estonia Medical Centre, 13419 Tallin, Estonia; 7 Department of Immunology and Allergology, Lithuanian University of Health Sciences, 50161 Kaunas, Lithuania; 8 North Estonia Medical Centre, Tallinn University of Technology, 13419 Tallinn, Estonia

**Keywords:** addiction, autoimmunity, cancer, cardiovascular diseases, cytokines, harm reduction, inflammation, nicotinic acetylcholine receptors, neurodegenerative diseases, smoking

## Abstract

Statistical data demonstrate a concurrent rise in smoking prevalence and mental disorders, such as depression and anxiety, which may be attributed to contemporary lifestyle factors, including social media and recent global events. This indicates a potential correlation between these trends, as individuals with mental disorders may engage in smoking as a form of self-medication to alleviate anxiety. However, smoking is harmful and increases the risk of many diseases. Therefore, smoking cessation strategies are increasingly being considered. Nicotine is a naturally produced alkaloid in plants that makes smoking so addictive. Unfortunately, the public’s lack of understanding of the effects of nicotine leads to misleading claims in the public and media about its biological effects. Thus, current narrative literature review is focused on the examination of the biological effects of organic nicotine from various angles, considering the psychological aspects of addiction and the immune system. Analysis of recent data showed that nicotine not only causes addiction but also has therapeutic benefits in certain diseases (depression, anxiety, schizophrenia) and has anti-inflammatory properties (autoimmunity, neurodegenerative diseases), and a deeper understanding and a broader approach to its effects is needed.

## 1. Introduction

### Psychological Aspects: Addiction and Behavior

Smoking remains a massive global issue, with over 1.3 billion users worldwide in 2023 (World Health Organization, WHO). The number of deaths attributable to combustible cigarette use has reached approximately 8 million annually, including many non-smokers affected by second-hand smoke [1]. Statistics show that not only the number of smokers is rising, but in parallel, the number of people suffering from mental disorders (depression or anxiety), which may have been caused by modern lifestyle (social media) and the challenges of recent years events around the world (e.g., war, COVID-19 pandemic) [2,3,4,5]. Depressive disorder cases grew from about 182 million in 1990 to 290 million in 2019 [6]. From 1990 to 2019, the global number of anxiety disorder cases increased by 73.44%, with age-standardized incidence rates rising by 21.17% [5]. In the first year of the COVID-19 pandemic, the global prevalence of anxiety and depression increased by a massive 25% [7]. Thus, the growth rate of these two factors, smoking and mental disorders, can be related.

Unlike other branches of medicine, psychiatrists and psychologists can view substances from the perspective of self-medication. They found that individuals with mental disorders use cigarettes as a self-treatment to manage anxiety, and studies have shown that smoking rates are significantly higher among individuals with anxiety and depression disorders [6,8,9,10,11,12]. Schizophrenics smoke heavily perhaps to self-treat their difficulties in gating repetitive auditory and visual stimuli [13]. From a scientific perspective, the relationship between smoking and anxiety could be explained by three possible non-mutually exclusive models [14]. First, smoking may contribute to increased anxiety; second, anxiety may drive higher smoking rates; and third, both smoking and anxiety may stem from shared vulnerability factors [15,16,17,18]. The same model could be used to explain depression’s relationship with smoking. However, there is no consensus on whether smoking increases or decreases symptoms of depression or anxiety, as the connection between smoking and anxiety is complex, and research results are controversial [16,19,20,21,22,23,24]. However, many smokers state that smoking is calming and quitting makes them feel more anxious [25]. Although smoking increases the sense of satisfaction in these individuals, at the same time, it is addictive.

Nicotine is a natural alkaloid found in several plant species (tomatoes, aubergines, and potatoes) but is at its highest level in tobacco plants [26]. It is addictive, with 85% of daily cigarette smokers being addicted to some extent, and even light smokers are at risk of addiction, with almost two-thirds of those smoking only 1–4 cigarettes per day being addicted [27]. Nicotine rapidly activates the brain’s reward circuits by triggering dopamine release. Over time, nicotine use leads to changes in brain structure and function, particularly in areas related to reward processing and executive control [28]. These neuroadaptations contribute to the development of tolerance and withdrawal symptoms, making quitting difficult. It is not common to refer to smokers as dependent individuals, even though diagnostic criteria clearly define it as an addiction. A significant change occurred with the release of the new diagnostic manual the International Classification of Diseases, 11th Revision (ICD-11), which revised the old definition of Tobacco dependence and transitioned to Nicotine dependence, emphasizing the role of nicotine the addictive substance—in this disorder [29]. ICD-11 shifts towards a more behaviorally focused definition of nicotine dependence, reducing emphasis on physiological dependence (withdrawal/tolerance) and instead highlighting compulsive use and loss of control. This update helps align the classification of nicotine addiction with modern understanding and treatment approaches while also encouraging further research on nicotine itself.

Looking at nicotine consumption from an addiction perspective, we recognize that it is a chronic relapsing disorder, where the primary goal is remission, but only a tiny percentage of users around 3–5% achieve remission on their own. However, even with the most effective interventions—combined pharmacotherapy and behavioral therapy—only about 24% of individuals achieve short-term remission lasting up to 12 months [30]. Furthermore, Dr. Nora Volkow, Director of the National Institute on Drug Abuse, highlights that abstinence is a pretty challenging goal to reach [31]. Only a few percent of individuals achieve it at once. Others may eventually achieve abstinence with repeated treatment, which could take years. In the process, we should identify treatments that would improve patients’ outcomes and reduce the risk of other adverse medical effects [31]. It is well-established that individuals with addictive tendencies frequently replace one addictive behavior with another, because different addictive substances work through a common dopaminergic pathway. This phenomenon suggests that forcing such a person to achieve abstinence may result in the reverse: the person may not break the primary addiction, and the risk of the emergence of possible replacement behaviors that will match similar psychological and neurochemical functions will remain [32]. In the context of smoking cessation, such substitutions may be compounds more harmful than nicotine, for instance, other stimulants or depressants, which can result in more severe social consequences—such as divorce, bankruptcy, or criminal behavior that may lead to social exclusion or impaired functioning.

Addiction is usually viewed through the lens of behavioral and psychological perspectives, but it also plays a significant role in the body’s physiology, especially concerning inflammation. Substance use affects the immune system, disrupts the natural balance of pro-inflammatory and anti-inflammatory cytokines, and contributes to long-term inflammatory responses that can complicate both active addiction and health recovery [33]. Unfortunately, the public lacks an understanding of the health effects of substances in tobacco products. Misconceptions about nicotine’s role in health risks are prevalent. Therefore, a comprehensive and multifaceted assessment of the biological effects of nicotine is helpful to ensure accurate and interpretable data. Given the trends that the combustible tobacco market is being replaced by several types of alternatives to cigarettes, which consist of one common substance–nicotine and the fact that users, medical society, and experts of regulators need to understand nicotine’s mechanism of action to evaluate its biological effects correctly, current review aims to analyze nicotine from a psychoneuroimmunological point of view, i.e., as a substance that affects behavior (addiction), the nervous system (neurotransmitters, receptors), and the immune response (inflammatory processes). As a result, the effects of nicotine are viewed from various angles: role in inflammation, autoimmunity, neurodegenerative, cardiovascular diseases and cancer.

## 2. Understanding Nicotine as a Substance

### 2.1. Neurobiological Aspects: Nicotinic Receptors

Nicotine is a chiral molecule with two stereoisomers, (S)-nicotine and (R)-nicotine. Natural nicotine from tobacco leaf contains > 99% (S)-nicotine, while synthetic nicotine is often a mixture of (R)- and (S)-isomers (usually 50% and 50%), as the cost of producing ultra-pure (S)-nicotine is very high [34,35]. Nicotine is absorbed most rapidly through the lungs (10–20 s), more slowly and evenly through the mucous membranes of the mouth (5–30 min), and through the skin (1–2 h), where it is absorbed readily and without peaking effects, before reaching the brain, where it acts on the nicotinic cholinergic receptors (a type of acetylcholine receptor, nAChRs) [36]. This results in the release of dopamine, which mediates the pleasurable experience of smoking [37]. Nicotine is primarily metabolized in the liver by three pathways: P450-catalyzed 5′-oxidation, UGT-catalyzed N-glucuronidation, and flavin monooxygenase catalyzed N′-oxidation to cotinine, which is the stable metabolite used to assess nicotine exposure [37,38].

Nicotine is structurally similar to the endogenous neurotransmitter acetylcholine (ACh). (S)-nicotine selectively binds to nAChRs. Nicotine binds to a site on the external face of the receptor and activates the release of various neurotransmitters, including catecholamines. An important property of nicotinic receptors is desensitization, which results in the development of acute tolerance. In the presence of nicotine, nAChRs transition to an inactive state and cannot be reactivated by nicotine [39,40]. (R)-nicotine has a lower affinity for the nAChRs. Therefore, the biological effects of synthetic nicotine, when in rancemic form, are weaker [41].

The nAChR complex is a pentameric ligand-gated ion channel with a central water-filled pore in neuronal and non-neuronal cells. It is formed by the neuronal subunits which are classified into alpha (α2–α7, α9, and α10) and beta (β2–β4) types, with the distinction based on the presence of adjacent cysteine residues in the extracellular domain, a feature unique to the alpha subunits [39,40,41,42,43]. α4β2 and α7 are the predominant subunit subtypes in the human brain. The former subtype has a high affinity to nicotine; therefore, the activation of α4β2 nAChRs by nicotine contributes to the development of addiction [44,45,46]. The α3β4 are commonly found in the peripheral nervous system, have a low affinity for nicotine, and have much slower desensitization kinetics than α4β2 nAChRs [43]. α7 of nAChR are also abundant in some non-neuronal cells, including microglia and macrophages. α7 of nAChR stimulation is associated with an anti-inflammatory effects, cell proliferation, angiogenesis, and resistance to drug-induced apoptosis [47]. There is data that nicotine’s interaction with the α7 subunit inhibits the cough reflex. A single-session exploratory analysis with healthy non-smokers showed dual action of nicotine: an immediate, peripheral protussive effect and a delayed central antitussive effect [48,49]. Hence, nAChRs have varied functions depending on where they are located within the body. In neural tissues, they are essential for cell growth, cognition, and addiction. In non-neuronal tissues, nAChRs are involved in multiple functions, such as inflammation, immune response, and the regulation of cell growth [50,51].

### 2.2. Immunological Response: Inflammation and Systemic Regulation

Understanding the mechanism of inflammation is necessary to understand the effects of nicotine on the body. Inflammation is a complex process involving multiple genes and signaling pathways [52]. There are several biomarkers used for the identification of inflammation such as cytokines (signaling molecules that modulate immune response): tumor necrosis factor-α (TNF-α), interleukin-1 (IL-1), IL-6, IL-8, or other enzymes and proteins, such as cyclooxygenase-2 (COX-2), 5-lipoxygenase (5-LOX), matrix metalloproteinases (MMP), C-reactive proteins, vascular endothelial growth factor (VEGF) and others [53]. However, IL-6 is often stimulated together with the pro-inflammatory cytokines TNF-α and IL-1 in many alarm conditions; IL-6 plays an important role in both local and systemic acute inflammatory responses by controlling the level of pro-inflammatory cytokines [54]. Elevations of cytokines IL-1β and IL-6 are known to be associated with the risk of atherosclerosis [53,55]. The cytokine spectrum can help identify the origin of inflammation. For example, general inflammatory diseases caused by an immune system disorder will elevate the cytokines TNF-α, IL-6, IL-8, and IL-12 [56]. Inflammation caused by lung, gastric, or breast cancer will be usually illustrated by elevation of cytokines TNF-α, IL-6, IL-8, interferon-γ (INF- γ) and several other cytokines [57,58,59]. The same cytokines are increased, except INF-γ, which is at a normal level in smoking induced inflammation [60]. On the contrary-anti-inflammatory ILs, such as IL-10 (inhibits monocyte/macrophage and neutrophil cytokine production and T helper I (Th1) type lymphocyte responses) and transforming growth factor-beta (TGF-β) (inhibits monocyte/macrophage MHC, class II expression and pro-inflammatory cytokines synthesis) play an important role in limiting inflammation and preventing tissue damage. Other anti-inflammatory cytokines include IL-4 (promotes Th2 lymphocyte development), IL-11 (inhibits pro-inflammatory cytokines response by monocyte/macrophages and promotes Th2 lymphocyte response), and IL-13 [61,62]. The interaction between pro-inflammatory and anti-inflammatory cytokines is an intricate and dynamic mechanism that shapes immune responses and maintains immune homeostasis.

### 2.3. Nicotine Effects

#### 2.3.1. Inflammation

It is still not precisely known whether nicotine has a pro-inflammatory or anti-inflammatory impact on the body because it is usually related to smoking. Therefore, it is difficult to separate if the effect is due to nicotine per se, as many constituents in cigarette smoke could be at the origin of the effect observed [63,64]. Nicotine increases the secretion of pro-inflammatory phase enzymes such as caspase-1 and cytokines such as IL-1β and IL-18 [65]. Furthermore, systemic immune effects in individuals who smoke also showed increased levels of reactive oxygen species (ROS) and an excess of circulating pro-inflammatory cytokines, including TNF-α and IL-6. Cytokine-induced chemokine release attracts neutrophils and leukocytes, amplifying the inflammatory response. Additionally, nicotine directly activates mast cells, triggering the release of pro-inflammatory mediators such as histamine and leukotrienes [66].

Although some of its effects are pro-inflammatory, nicotine effects are mostly anti-inflammatory [67]. Nicotine suppresses the innate and adaptive immune response by reducing the secretion of pro-inflammatory cytokines (IL-1, IL-6, TNF-α), which reduces the activity of lymphocytes and decreases the possibility of cytokine storm and sepsis [68]. The cytokine storm is a dangerous hyperinflammatory state associated with elevated levels of several pro-inflammatory cytokines (IL-1β, IL-2, IL-6, IL-17, IL-8, TNF-α) and chemokine L2 (CCL2) and it is an integral part of sepsis. In a model of sepsis, nicotine inhibits Toll-like receptor 4 (TLR4) (LPS)-mediated inflammation and decreases the production of pro-inflammatory cytokines, effectively increasing survival via α7-nAChR [63]. Nicotine α7 receptor appears to be expressed on CD4^+^ lymphocytes, and stimulation with nicotine in these receptors reduces proliferation and activation of T-cells. All of these events decrease the secretion of pro-inflammatory cytokines: IL-17, IL-21, and IL-22 [65]. On the other hand, nicotine suppresses the activation of dendritic cells, which are important for the induction, differentiation, and expansion of Th1 cells. As a result, cell-mediated immunity against infection and neoplastic diseases is downregulated [69].

#### 2.3.2. Autoimmune Diseases

The probability that nicotine exerts more anti-inflammatory effects than pro-inflammatory ones is associated with beneficial effects in some cases of autoimmune diseases. For example, in vitro and animal studies showed that nicotine reduces T cell receptor (TCR) signaling and suppresses the production and secretion of antibodies, potentially improving the condition of ulcerative colitis by maintaining intestinal barrier integrity [67,70]. Rheumatoid arthritis is an autoimmune disease with imbalance polarization toward Th1 and Th2 that produces cytokines (IL-1β, IL-6, IL-17, TNF-α, IFN-γ, and IL-8) causing inflammation, which destroys the joints. Animal studies showed that nicotine promotes anti-inflammatory activity and disease modulation by decreasing TNF-α and promoting Th2 immune response [67].

#### 2.3.3. Cancer Development

There is no evidence that nicotine itself provokes cancer [37,67,71,72]. Nicotine is not identified as a carcinogen in the list of the International Agency for Research on Cancer (IARC) by WHO as well [73]. However, some of its metabolites can form low levels of carcinogenic compounds under specific conditions (e.g., nitrosation) [74]. The adverse effects of nicotine in cancer patients are linked to its impact on the immune system components. For instance, nicotine activates several types of cytokines and chemokines, which may increase angiogenesis and suppress cancer cell apoptosis, thus exacerbating tumors due to increasing survival and cell growth [63]. Therefore, in patients who already have cancer, nicotine exposure can reduce the effectiveness of anti-cancer treatment. In non-neuronal cells, neurotransmitters (like adrenaline and noradrenaline) play a role in the growth of various cancer types of cells by directly activating intracellular signaling pathways (PKC, AKT, ERK) or indirectly by releasing factors like epidermal growth factor (EGF) and vascular endothelial growth factor (VEGF), which regulate proliferation, migration, and angiogenesis [35,42]. Studies with mouse models demonstrated that nicotine generates a pro-inflammatory microenvironment in the lungs characterized by an influx of activated neutrophils, which results in a favorable premetastatic niche [75]. On the other hand, there is data about nicotine’s inhibitory effect on the proliferation of ovarian cancer cells through the nAChR α4, α5, α7 subunits, which are expressed in ovarian cancer cells [76]. Thus, nicotine may enhance or inhibit already existing cancer cell proliferation depending on the type of cancer.

#### 2.3.4. Neurodegenerative Diseases

After evidence of nicotine’s effects on neurodegenerative diseases such as Alzheimer’s and Parkinson’s has accumulated, its medical value has received considerable attention due to its beneficial anti-inflammatory, anti-apoptotic, pro-cognitive, and anti-protein aggregation effects in neurodegenerative diseases [77]. Nicotine slows the progression of Parkinson’s disease by inhibiting the action of the stress-responsive protein deacetylase sirtuin 6, thereby reducing neuronal apoptosis and improving neuronal survival. In Alzheimer’s disease, nicotine improves cognitive impairment by increasing the activity of protein kinase B (also known as Akt) and stimulating phosphoinositide 3-kinase/Akt signaling, which regulates learning and memory processes [78]. The results of clinical trials with subjects with mild cognitive impairment showed nicotine-induced cognitive improvement test results after 6 months of transdermal nicotine use [79].

Nicotine may also activate thyroid receptor signaling pathways, improving memory impairment caused by hypothyroidism in that way. In healthy individuals, nicotine improves memory impairment caused by sleep deprivation by enhancing the phosphorylation of calmodulin-dependent protein kinase II, an essential regulator of cell proliferation and synaptic plasticity and function through its effect on chromatin modification via the inhibition of histone deacetylases, which causes transcriptional changes in memory-related genes. Finally, nicotine administration has long-term modulatory effects in individuals with chronic stress, hypothyroidism, schizophrenia, stress-induced anxiety, and depression, which may be attributed to its dual action on nicotinic acetylcholine receptors—namely, initial stimulation of α7 receptors (which is particularly relevant to sensory gating and cognitive modulation), followed by receptor desensitization that may contribute to downstream adaptive or regulatory processes [77,80]. The evidence from human studies remains contradictory: some studies show cognitive benefits [81,82], while others find no significant impact, and neither reveals any apparent adverse effects [83]. Therefore, further research is needed to determine the safety of nicotine treatment and its long-term effects.

#### 2.3.5. Cardiovascular Diseases

Cardiovascular disorders (CVDs) are a worldwide problem and are considered to be one of the leading causes of mortality. It was found that inflammation has a crucial role in the pathogenesis of these disorders due to the accelerated atherosclerotic process. The link between TNF-α, ILs such as IL-1, IL-6, IL-12 and systemic inflammatory conditions with CVDs, including acute myocardial infarction (AMI), chronic heart failure (CHF), venous thromboembolism (VTE) and others was identified [12,84]. During chronic stress also in people suffering from depression or anxiety, levels of the same cytokines (IL-1, IL-6) are elevated, leading to chronic inflammation [85,86]. Regard that around 28–40% [86,87] of people with depression or anxiety smoke and that both the disease itself and smoking contribute to chronic inflammation, it makes it clear that the impact on the cardiovascular system is complex and that it is difficult to separate the effect of a single factor. It is even harder to distinguish the impact of nicotine itself from tobacco combustion products on the incidence of cardiovascular diseases.

The effect of nicotine on the cardiovascular system can be assessed from two perspectives: after acute and chronic exposure. Primarily, nicotine stimulates the sympathetic nervous system, releasing norepinephrine, which increases the heart rate, blood pressure, myocardial contractility, and systemic vasoconstriction, and blood flow in the skeletal muscles. This effect lasts about 20 min (after smoking) [88]. However, the exact vascular effects of nicotine and the underlying mechanisms are not clarified yet because, evaluating the effects of nicotine itself properly, we cannot rely on the results of studies that analyze the health effects of smoking [89]. From a chronic exposure perspective, studies with nicotine medications did not show an increased risk of cardiovascular events in patients given nicotine medication in subjects with acute and chronic cardiovascular diseases [90]. For example, real-life studies did not identify a statistically or clinically significant association between the use of nicotine patches and myocardial infarction in smokers without known cardiovascular diseases. Furthermore, there was no connection between transdermal nicotine use and a significant increase in cardiovascular events in high-risk outpatients with cardiac disease either. Even though nicotine ‘s pharmacologic effects may contribute to cardiotoxicity, it seems that transdermal nicotine therapy is likely to be less dangerous to patients with cardiovascular diseases than smoking because it does not increase thrombogenesis, decrease oxygen-carrying capacity, or lead to atherogenesis [91,92,93,94]. According to studies with patients with coronary artery disease results, combustion tobacco products rather than nicotine were the primary contributors to smoking-induced cardiac ischemia. Such conclusions were reached when subjects started to use nicotine patches and reduced combustible tobacco consumption [95]. The fact that combustion products, not nicotine, are associated with cardiac ischemia was reported by the study with heated tobacco products (HTPs), which showed that switching from cigarettes to HTPs for one month resulted in improved endothelial function, oxidative stress burden as well as in the reduction of platelet activity and exposure to CO, while caused an improvement in coronary flow reserve and myocardial work efficiency compared with cigarette smoking [96]. Such results suggest that nicotine itself is less associated with the likelihood of cardiovascular disease in individuals who are not at increased risk.

#### 2.3.6. Respiratory Diseases

Direct human studies evaluating the impact of nicotine on respiratory health are scarce. The effect on the airways of nicotine is often mistaken for those of tobacco smoke. However, it is necessary to distinguish between the composition and effects of pure nicotine and tobacco smoke. Tobacco smoke consists of more than 7000 chemical compounds, including carcinogens and irritants that directly affect the respiratory tract, and nicotine is not the major cause of the harmful effects [97,98,99]. In vitro studies showed that nicotine affects the IL-22 axis, inhibiting the production of IL-22 by T cells, thereby weakening the immune barrier of lung epithelial cells against infection [49,100]. Furthermore, studies with airway epithelial cells have shown that nicotine can stimulate the expression of pro-inflammatory cytokines (e.g., IL-6, IL-8, TNF-α) in the airways. It also affects ciliary function by reducing mucus clearance through inhibition of CFTR (Cystic Fibrosis Transmembrane Conductance Regulator) channels [101].

However, clinical data on the effect of nicotine as the sole active ingredient on the development of COPD is limited. Existing studies are primarily based on in vitro models, animal studies, or exposure to e-cigarette aerosol (containing nicotine), so caution is warranted when applying the results to human physiology.

Table 1 presents the nicotine effect on the human body via biological pathways.

## 3. Discussion

The effects of nicotine on the human body are multifaceted and can only be fully understood by integrating mental, neurological, and immunological mechanisms. The psychoneuroimmunological analysis provides insight into how addictive behavior (dopaminergic system), the nervous system (nAChR), and inflammatory processes (cytokine changes) form an interconnected web of responses. Accurate assessment of nicotine’s effects on human health is challenging because it is hard to distinguish its effects from other factors (smoking). In addition, very few studies have been conducted on humans, so the results remain controversial. It should be understood that nicotine acts on receptors that are normally activated by endogenous acetylcholine. The cholinergic nervous system regulates major physiological functions by interacting with many other modulating factors and feedback mechanisms. The fact that nicotine exerts its effects in an isolated in vitro test system does not prove that the same effects will occur in vivo under normal homeostatic mechanisms. Many nicotine-induced responses are characterized by a complex dose-response relationship, with low doses activating and higher doses inhibiting responses. Therefore, the doses of nicotine used in pre-clinical studies must be carefully evaluated to predict its effects on humans adequately [40,102]. Nicotine’s health effects are explained through a complex and intricate mechanism of inflammation that is influenced by many other factors so that the effects of nicotine itself on people’s health can only be presumed.

About 27% of subjects with cardiovascular diseases are current smokers [87]. Such a fact shows that doctors’ advice to quit smoking, with all the negative consequences of not doing so, is an ineffective way to reduce smoking rates. This is a result of nicotine being addictive. Given the highest number of dependent individuals compared to other substance or behavioral addictions, the need for primary treatment of co-occurring mental health disorders, and the relatively low probability of long-term remission, we need to seek alternative approaches and consider harm reduction concepts. This may involve guiding smokers of combustible cigarettes toward other non-combustible nicotine-containing products, thereby reducing potential harm to the body while simultaneously satisfying nicotine cravings as an intermediate step toward complete abstinence. The most successful treatment combines pharmacotherapy and behavioral therapies, allowing 24% of those who quit smoking to remain abstinent for up to 12 months [30]. According to the American Society of Addiction Medicine (ASAM) guidelines, redirection of ambivalent patients toward alternative nicotine products—which are not harmless but, according to preliminary research, cause less harm to organs while still satisfying the nicotine need of dependent individuals could be beneficial. Studies to date suggest that new substitute non-combustible tobacco products may have the potential to reduce smokers’ population health risks compared to conventional cigarettes and could be an alternative to minimize the risk associated with cigarette smoking [103,104,105], thereby reducing potential harm to the body while simultaneously satisfying nicotine cravings as an intermediate step toward complete abstinence. Although alternatives to combustible cigarettes are not entirely risk-free, evidence suggests that they pose a lower health risk compared to combustible tobacco products among adult smokers who are unable to quit. However, long-term safety is unclear, clinical data are insufficient but given that we are dealing with a population of smokers whose behavior does not change (they don’t want to quit or can’t), the interim predictive emission-exposure data available to date suggest a potential for reduced harm [105,106,107].

There are several types of alternatives to cigarettes, but all of them consist of one common substance-nicotine [108]. However, the public dissemination and interpretation of scientific research results is fraught with misinformation that confuses smokers about the effects of certain substances on human health. As a result, the concept of tobacco risk reduction itself is viewed in a false negative manner [109,110]. Independent research is therefore needed to identify evidence-based products that are less harmful to the health of people who are unable to quit smoking.

## 4. Conclusions

The current review of literature shows that, as a substance itself, organic nicotine is a natural compound with multiple effects on the human body, which are related to its inflammatory and anti-inflammatory properties, without a carcinogenic direct impact. Traditionally, the influence of nicotine is misleadingly equated with smoking. However, studies have shown that it may have a positive effect in several neurological or psychiatric conditions (anxiolytic properties, improves cognitive functions) and modifies the disease course during autoimmunity. Despite nicotine having potential therapeutic properties, it may also cause addiction. However, it does not cause negative social consequences (e.g., divorce, bankruptcy, or criminal behavior that may lead to social exclusion or impaired functioning) compared with other psychoactive substances (alcohol, heroin, cocaine, methamphetamine). Given that smoking may cause an addictive disease and long-term remission, like other addictions, is an elusive goal, the application of harm reduction principles could be considered for ambivalent patients. Less harmful systems satisfying addiction without smoking-associated health risks could be a pragmatic and future-oriented strategy for managing nicotine use and addiction.

## Figures and Tables

**Table 1:** Summarizing table of nicotine’s effect on the human body via biological pathways (according to human studies). ↑—indicates an increase, ↓—indicates a decrease of production/activation.

The Group of Diseases	Pathway	Biological Effect	References
Autoimmunity	↓ TNF-α ↑ T helper II (Th2)	Improves the condition of ulcerative colitis and rheumatoid arthritis.	[67,70]
Cancer development	↑ several types of cytokines and chemokines, which may increase angiogenesis and suppress cancer cell apoptosis ↑ intracellular signaling pathways (PKC, AKT, ERK) or ↑ factors like epidermal growth factor (EGF) and vascular endothelial growth factor (VEGF), indirectly	Is not identified as a carcinogen and does not cause cancer; Creates an immunosuppressive environment in patients who already have cancer complicating anti-cancer treatment.	[34,37,40,64,69,70]
Neurodegenerative diseases	↓ protein deacetylase sirtuin 6 ↑ thyroid receptor signaling pathways ↑ activity of protein kinase B (Akt) and phosphoinositide 3-kinase/Akt signaling ↑ phosphorylation of calmodulin-dependent protein kinase II	Slows the progression of Parkinson’s and Alzheimer‘s diseases; Improves memory in healthy adults and patients with mental disorders.	[74,75,76]
Cardiovascular diseases	↓ IL-1 ↓ IL-6 ↑ sympathetic nervous system, contractility, and systemic vasoconstriction, and blood flow in the skeletal muscles	Short term effect: releases norepinephrine, which increases the heart rate, blood pressure, myocardial contractility, and systemic vasoconstriction, and blood flow in the skeletal muscles. Long term effect: does not show risk for healthy subjects. In vitro: stimulates the morphogenesis of fibroblasts into a myofibroblastic phenotype.	[81,83,84,86,87,89]
Respiratory diseases	↓ IL-22 ↑ IL-8, IL-6, TNF-α	No data from human studies; In vitro: decreased lung epithelial barrier function.	[46,92]

## Data Availability

Not applicable.

## Abbreviations

The following abbreviations are used in this manuscript

CVDs	Cardiovascular disorders
ILs	Interleukins
nAChRs	Nicotinic acetylcholine receptors
Th	T helper cells
WHO	World Health Organization
